# Rational Diversification of a Promoter Providing Fine-Tuned Expression and Orthogonal Regulation for Synthetic Biology

**DOI:** 10.1371/journal.pone.0033279

**Published:** 2012-03-19

**Authors:** Benjamin A. Blount, Tim Weenink, Serge Vasylechko, Tom Ellis

**Affiliations:** 1 Centre for Synthetic Biology and Innovation, Imperial College London, London, United Kingdom; 2 Department of Bioengineering, Imperial College London, London, United Kingdom; University of Kent, United Kingdom

## Abstract

Yeast is an ideal organism for the development and application of synthetic biology, yet there remain relatively few well-characterised biological parts suitable for precise engineering of this chassis. In order to address this current need, we present here a strategy that takes a single biological part, a promoter, and re-engineers it to produce a fine-graded output range promoter library and new regulated promoters desirable for orthogonal synthetic biology applications. A highly constitutive *Saccharomyces cerevisiae* promoter, *PFY1*p, was identified by bioinformatic approaches, characterised *in vivo* and diversified at its core sequence to create a 36-member promoter library. TetR regulation was introduced into *PFY1*p to create a synthetic inducible promoter (i*PFY1*p) that functions in an inverter device. Orthogonal and scalable regulation of synthetic promoters was then demonstrated for the first time using customisable Transcription Activator-Like Effectors (TALEs) modified and designed to act as orthogonal repressors for specific *PFY1*-based promoters. The ability to diversify a promoter at its core sequences and then independently target Transcription Activator-Like Orthogonal Repressors (TALORs) to virtually any of these sequences shows great promise toward the design and construction of future synthetic gene networks that encode complex “multi-wire” logic functions.

## Introduction

Synthetic biology aims to use modular, well-characterised biological parts to predictably construct novel genetic devices and complex cell-based systems following engineering principles [1]. This process has shown particular promise in *Escherichia coli*, where many useful parts and devices have been described [2], [3] and a public-benefit facility (biofab.org) now exists to produce free-to-access collections of standard biological parts [4]. Rationally combining well-characterised parts and devices using modelling to aid design has allowed complex systems to be produced, endowing bacteria with novel abilities such as pattern formation [5], edge-detection [6] and co-ordinated oscillation [7]. Key parts that enable this advanced engineering include promoters, particularly regulated promoters, as these control gene network logic and are routinely used to provide nodes at which to combine devices [6]. Currently, there are a limited number of well-characterised promoter systems in synthetic biology and this presents a bottleneck to increasing the complexity of designs [8]–[10].

The budding yeast *Saccharomyces cerevisiae* has a long history of use in biotechnology and has also emerged as a versatile chassis for synthetic biology. Yeast cells have been previously engineered using synthetic biology to exhibit a variety of useful properties that include regulatory responses to light as an input, production of a precursor of the anti-malarial drug artemisinin, and expression of external cellulosome components that allow cellulose degradation [11]–[14]. It has also been demonstrated that model-based approaches can be used to investigate and produce predictable behaviours in synthetic gene networks in yeast [15], [16]. Furthermore, recent ground-breaking work placing synthetic re-factored chromosomal arms into living cells indicates great promise for the future construction of large, modular synthetic biology systems in yeast [17]. Yet, to accelerate yeast to a chassis for advanced synthetic biology on par with *E. coli*, there is still a need for more fundamental parts and devices, particularly as multiple re-use of the same biological parts in *Saccharomyces* poses problems due to the cell's natural ability to recombine long stretches of homologous DNA [18]. An attractive strategy to tackle this lack of parts is to diversify simple, well-characterised natural parts to obtain new synthetic ones with desired properties. Previous work has demonstrated that yeast promoters are suited to diversification [16], [19]–[22] and given their fundamental importance in synthetic biology, schemes to rationally modify promoters for new regulation properties and fine-tuned output are valuable for advancing research and applications.

While synthetic gene networks with predictable behaviour have been realised in *S. cerevisiae* [15], [16], [23], the complexity of such devices is likely to be limited by the number of available promoters that can be independently regulated. The majority of natural promoters in yeast are regulated by the cell's own transcription factors, and so to establish predictable synthetic gene networks within this requires new transcription factors that control gene expression independently, effectively acting as new insulated wires between parts. In synthetic biology, this property is typically called ‘orthogonality’ [24]. Orthogonality primarily provides related yet independent parts for synthetic networks thus avoiding network cross-talk in engineered systems. It also offers a further benefit by theoretically reducing interactions with endogenous networks within the host cell. The recent discovery and exploitation of transcription activator-like (TAL) effectors, found in plant pathogenic bacteria to positively regulate host gene expression, has allowed the rational design and construction of modular transcription factors that bind to specified DNA sequences [25]–[28]. These have been customised to perform genome editing in cells and to activate mammalian and plant gene expression but have yet to have been demonstrated as orthogonal repressors [29]. This new technology, combined with methods to diversify promoters to create synthetic libraries, offers the possibility of a scalable strategy for delivering fine-tuned and orthogonally-regulated promoters essential for yeast synthetic biology.

In this work we demonstrate our strategy by using available bioinformatics and *in vivo* characterisation to identify a short, simple promoter native to *S. cerevisiae* that can be rationally diversified to deliver new parts. We generate a synthetic promoter library by randomising core promoter sequences and then recode the promoter to be regulated by TetR, allowing construction of an inducible inverter device. Finally, we demonstrate that Transcription Activator-Like (TAL) proteins can be rationally constructed to bind independently to wild-type and recoded core promoters thus yielding user-defined orthogonal regulation by repression.

## Results

### Identification of a Candidate Constitutive Promoter

In order to obtain a yeast promoter suitable for rational reengineering, we sought to identify constitutive promoters with the minimal amount of natural regulation. To do this we took a bioinformatics approach, making use of a large number of datasets from published microarray experiments to identify candidate promoters whose output rarely varied despite different perturbations. To generate an initial shortlist of candidate genes, quantitative microarray expression data were taken from an industrially-relevant study conducted by Daran-Lapujade *et al.* where cells were grown in different carbon sources in a chemostat [30]. The average expression level of all genes across all conditions in this study was 271.2 and to eliminate genes with expression levels that may be too low to detect in synthetic devices, or that may stress the cell during overproduction, genes with average expression values lower than 200 or higher than 2000 were removed. Also removed were genes with no assigned function. The reduced list of 1163 genes was then ranked by coefficient of variation (CV) across the conditions and the 12 genes with the lowest values composed the shortlist of candidates. This shortlist comprised of *ALA1*, *ARA1*, *DBP5*, *GIM5*, *HRT1*, *MDH3*, *PFY1*, *RPL6A*, *RPN6*, *VMA6*, *YKT6* and *YSA1*.

**Figure 1:**
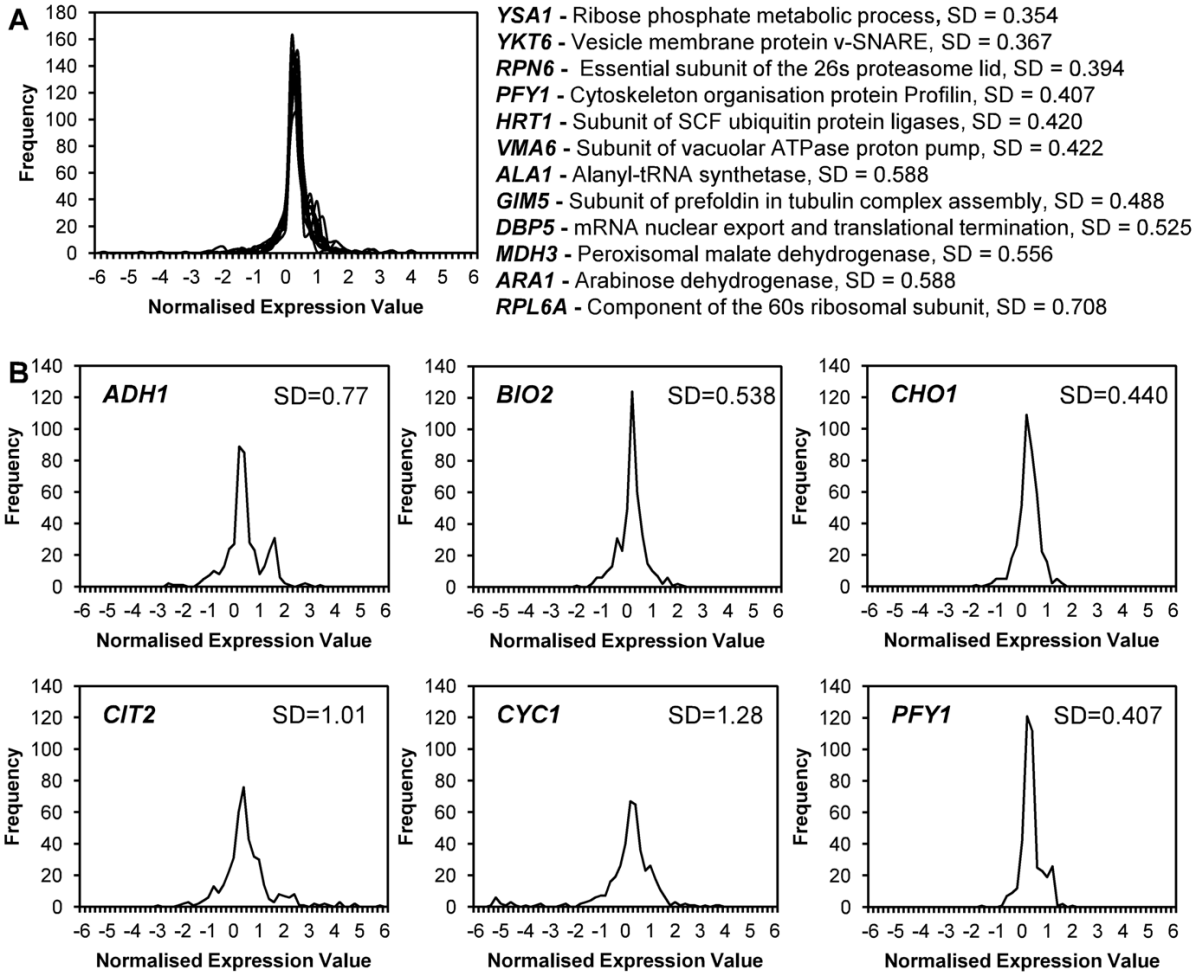
Analysis of existing *S. cerevisiae* expression data to identify constitutive promoters. Frequency of normalised expression values in selected microarray datasets taken from the SPELL database. (A) shows overlaid curves representing distribution of gene expression values (log_2_) across all of the selected datasets for all of the initial 12 candidate genes, a brief description of function and standard deviation (SD) of gene expression across the datasets is given for each gene individually. (B) shows the distribution of gene expression values and standard deviation across the datasets for the 6 genes with promoters that were experimentally characterised.

A total of 20 experimental microarray datasets from the Serial Pattern of Expression Levels Locator (SPELL) database were selected for their relevance to synthetic biology and encompassed multiple carbon sources, nitrogen sources, nutrient limitation, stress response, protein overexpression, fermentation, peroxisome induction, cell cycle and cell aging conditions [12], [31]–[44]. Expression data was collected from these datasets for each of the shortlisted genes using the YeastMine tool of the Saccharomyces Genome Database (yeastmine.yeastgenome.org) [45], [46]. In total there were 415 values, each corresponding to the normalised average expression level under a particular experimental condition. The distribution of expression levels was very similar for all of the candidate genes, with standard deviation (SD) across all the conditions ranging from 0.354 for *YSA1* to 0.708 for *RPL6A*, indicating that all of the genes are expressed to very similar levels under a wide variety of conditions (Fig. 1A). As all of the shortlisted genes showed robust expression, the promoter sequence chosen was that of the Profilin encoding gene *PFY1*, as this promoter has a relatively short sequence with minimal regulatory elements, had the fourth lowest standard deviation between the experimental conditions (0.407) and of the shortlisted genes had the most well-characterised promoter [47], [48].

To test the suitability of the *PFY1* promoter (*PFY1*p) as a promoter for synthetic biology, 5 other promoters were selected to characterise *in vivo* alongside it. The promoters chosen were the well-characterised *ADH1*p and *CYC1*p as well as *BIO2*p, *CHO1*p and *CIT2*p. SPELL-database analysis of the expression levels of the corresponding genes, using the same methodology as above, showed these promoters to represent a fairly large range of variability in expression, with SD values ranging from 0.440 for *CHO1* to 1.28 for *CYC1* (Fig. 1B).

### Characterisation of Yeast Promoters in a Synthetic Biology Context

**Figure 2:**
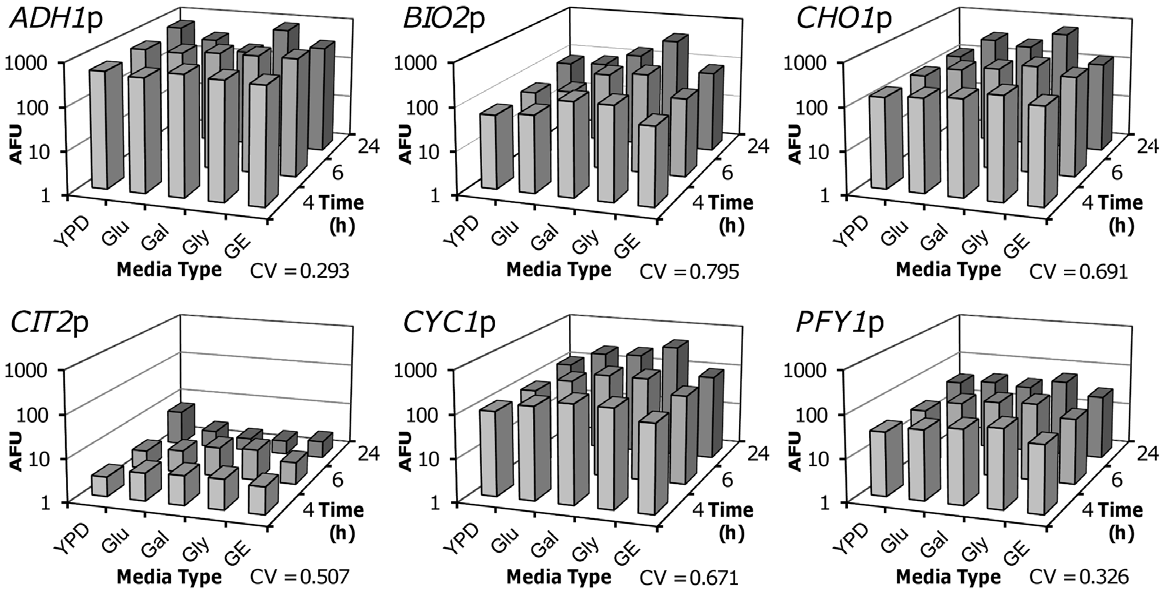
Characterisation of *PFY1*p against 5 other yeast promoters. Fluorescence output of chromosomal single-copy *yEGFP* under the control of *ADH1*p, *BIO2*p, *CHO1*p, *CIT2*p, *CYC1*p and *PFY1*p as determined by flow cytometry in triplicate. Fluorescence is displayed in arbitrary fluorescence units (AFU) and represents the mean average of the geometric mean values of three replicate cultures. Media types are YPD, and SC media with 2% glucose (Glu), galactose (Gal), glycerol (Gly) or glucose and ethanol (GE) as added carbon sources. Coefficient of variation (CV) is calculated using geometric mean values from each replicate under each media condition at each time point.

To characterise the selected promoters *in vivo*, each was cloned into a pRS406 derived vector upstream of an expression cassette consisting of a short 5′ untranslated region (UTR), a yeast-enhanced Green Fluorescent Protein (*yEGFP*) coding sequence and a *CYC1* terminator. This generated the yeast integrative plasmids pSV-*ADH1*p, pSV-*BIO2*p, pSV-*CHO1*p, pSV-*CIT2*p, pSV-*CYC1*p and pSV-*PFY1*p. As the only difference between these is in the promoter sequence, any variation due to context is minimised. A single copy of each was integrated into the YPH500 haploid strain genome [49] at the *URA3* locus of chromosome V. In triplicate, each promoter strain was then grown over a 24 hour time course in YPD and in synthetic complete (SC) media with different carbon sources at 2%, namely glucose, galactose, glycerol and glucose plus 2% ethanol. Single cell fluorescence levels were measured by flow cytometry at 4 and 6 hours, when the cultures were all displaying exponential growth and at 24 hours, when all of the cultures were at stationary phase (Fig. 2). The resultant data show that *PFY1*p has a moderate output under all conditions with a CV between all replicates of all conditions of 0.326. The only promoter tested with less variation was *ADH1*p, which had a CV of 0.293 but has been previously shown to be down-regulated under certain circumstances such as low oxygen conditions [50]. Of the other promoters characterised, all had CV values higher than 0.5 with *BIO2*p displaying high reproducibility between replicates but high variation between media types, *CHO1*p and *CYC1*p showing poor reproducibility between replicates and *CIT2*p having very weak output.

### Creation of a PFY1p-based Synthetic Promoter Library

Previous work has described the *PFY1* promoter as a minimal constitutive promoter that does not use a TATA-box mediated regulation mechanism and has no sequence-specific binding over its ∼100 bp core sequence. Immediately upstream of the core sequence it contains an rDNA enhancer-binding protein (Reb1P) binding site and a poly-dT element that are thought to maintain a constant bend in the *PFY1*p DNA that allows constitutive access for RNA polymerase complexes to initiate transcription [47]. As is typical of eukaryotic constitutive promoters, the transcriptional start site location appears to be variable across a 20 bp sequence at the end of the promoter [48], [51]–[54].

Whilst the Reb1P binding site and poly-dT elements of *PFY1*p are important to maintain stable and constitutive expression, other sequences in the core promoter region theoretically can be changed to generate promoters with different transcriptional properties. Directed mutation in this region will give DNA sequences that have altered melting characteristics and form different interactions with the RNA polymerase pre-initiation complex. This will lead to different transcription initiation efficiencies and thus with appropriate selection it is possible to generate promoter libraries that have a range of expression outputs.

**Figure 3:**
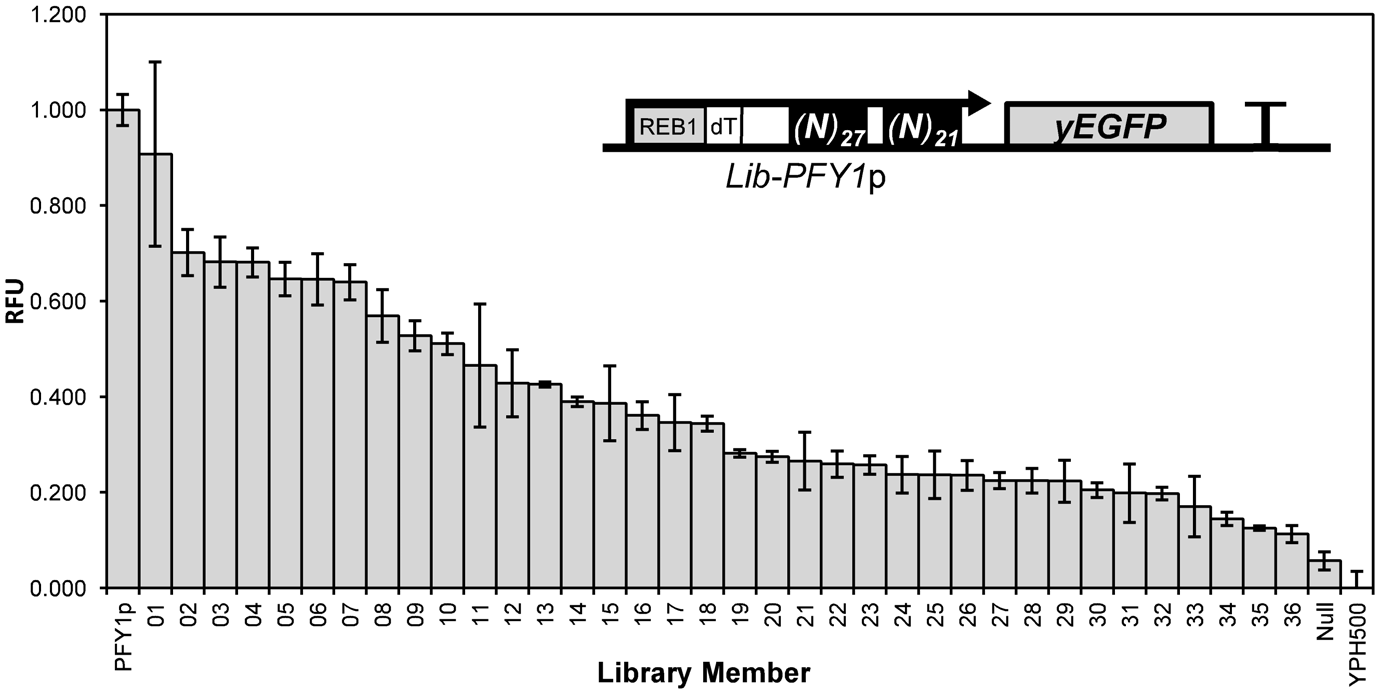
Characterisation of a *PFY1*p synthetic promoter library. Fluorescence output of synthetic library promoters driving expression of a single chromosomal copy of *yEGFP* as determined by flow cytometry in triplicate. Fluorescence is displayed in relative fluorescence units (RFU) and represents the mean average of geometric mean values as a proportion of a *PFY1*p control value. Error bars represent 1 standard deviation from the mean. The inlaid genetic circuit diagram shows the structure and context of the library promoters.

**Figure 4:**
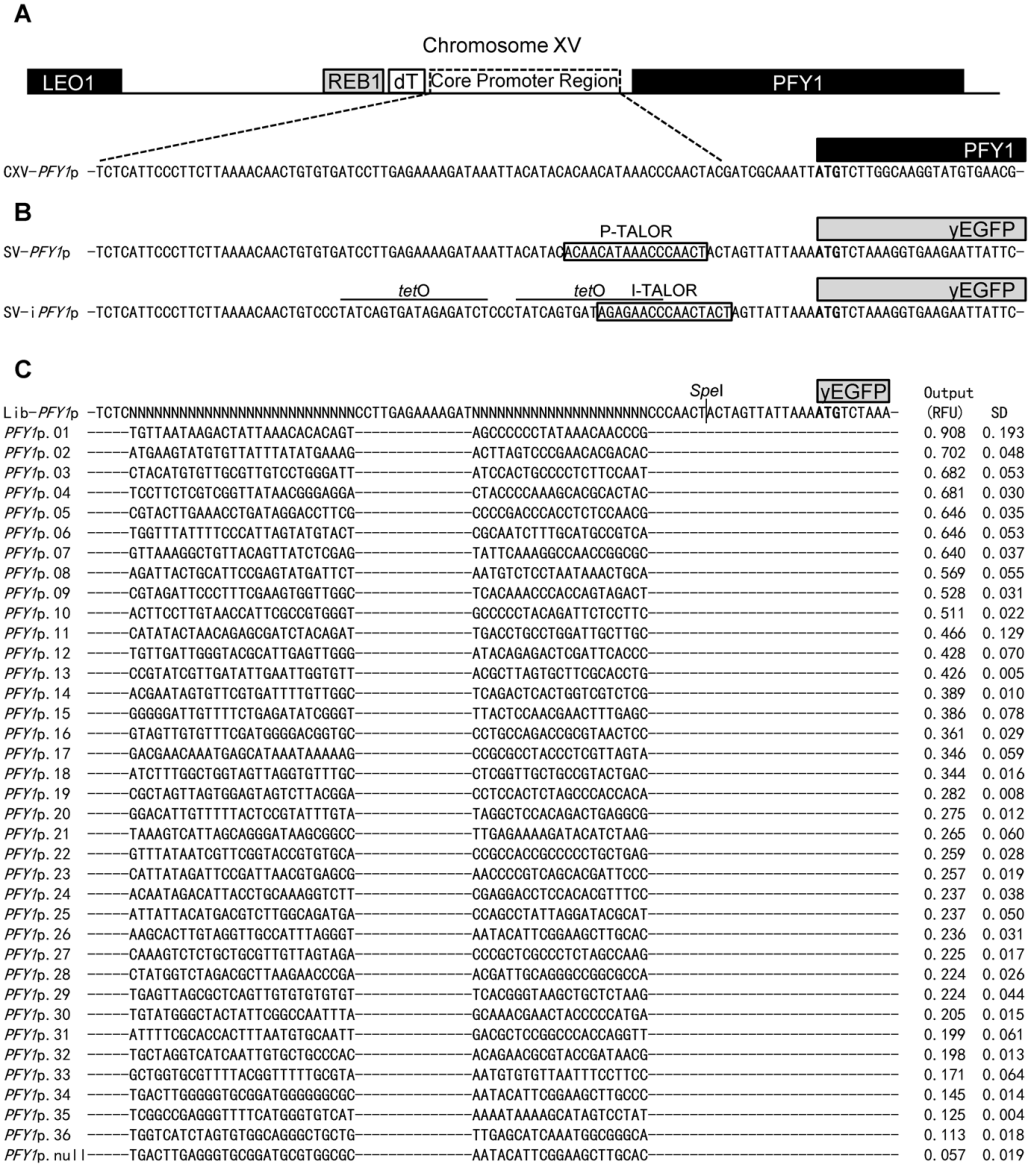
Overview of engineered promoter sequences. (A) shows the natural context and core promoter region sequence of *PFY1*p. Bases in bold represent the start codon. (B) shows *PFY1*p and i*PFY1*p core promoter region sequences in the context of the *yEGFP* expression cassette used for promoter characterisation. A vertical black line within the sequence represents a *Spe*I restriction enzyme cleavage site. P-TALOR and S-TALOR binding regions are highlighted with black boxes and *tet*O_2_ operator sequences are denoted by a horizontal black line above the sequences. (C) shows the variable sequence regions of members of the synthetic promoter library along with relative fluorescence unit (RFU) output and standard deviation (SD) values. Dashed horizontal lines denote sequence identity among library members.

To generate a *PFY1*p-based promoter library with 48 bp of the core promoter region randomised, we followed the synthetic promoter library method [55], [56], modifying it to include circular polymerase extension cloning (CPEC) [57] into the pSV-*PFY1*p plasmid. Following integration into the *URA3* locus and subsequent screening of colonies for fluorescence output by flow cytometry, we selected a clone with a null output and then a further 36 clones that expressed *yEGFP* with a graded-range of outputs. These promoters were sequenced, verified as single-integrants and characterised for expression output by flow cytometry measurement of yEGFP after 4 hours growth in SD media without uracil (Fig. 3 and Fig 4). The library members characterised range in output from 91% of *PFY1*p output down to 11% of *PFY1*p output. The promoter outputs are distributed fairly evenly so that when ranked by output, the biggest difference between neighbouring promoters is between *PFY1*p.01 (91% of *PFY1*p output) and *PFY1*p.02 (70% of *PFY1*p output) and the mean difference in output between neighbouring promoters is less than 2.5% of *PFY1*p output.

### Engineering TetR-Mediated Repression into PFY1p

**Figure 5:**
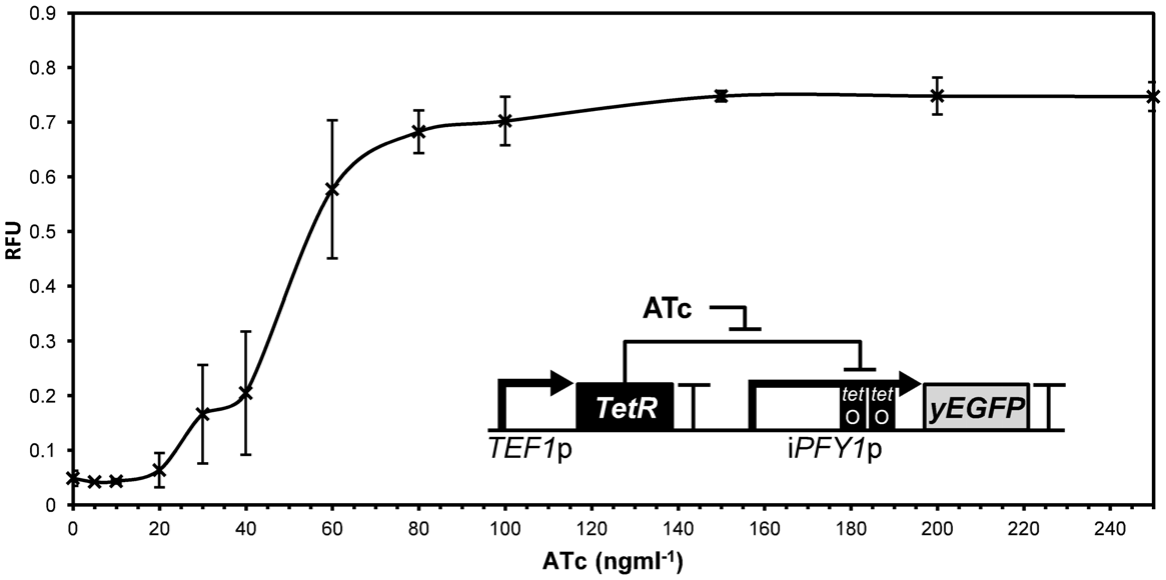
Response of i*PFY1*p-based inverter to anhydrotetracycline. Fluorescence output of i*PFY1*p in the pINV1 inverter as determined by flow cytometry in triplicate after 6 hours growth following induction with varied concentrations of anhydrotetracycline (ATc). Fluorescence is displayed in relative fluorescence units (RFU) and represents the mean average of geometric mean values as a proportion of a *PFY1*p control value. Error bars represent 1 standard deviation from the mean. The inlaid genetic circuit diagram shows the layout and function of the inverter.

Following the demonstration that major changes can be made to the *PFY1*p core sequence while maintaining measurable output, we next sought to rationally engineer regulation. In addition to fine control of output, the engineering of regulatory sites into a promoter is desirable for the construction of synthetic gene networks. The introduction of such sites into yeast promoters has been previously achieved, giving regulated promoter parts that enable the construction of logic functions [16], [19]. *PFY1*p was modified to display repression by TetR by introducing tandem TetR operator sequences at the core promoter region. The modified promoter, designed to be subjected to TetR-based inhibition was named i*PFY1*p and was hosted in integrative plasmid pSV-i*PFY1*p. In order to test this promoter and create a *PFY1*p-based inverter device, an expression cassette consisting of *TEF1*p-*tetR*-*ADH1* terminator was assembled and inserted into pSV-i*PFY1*p upstream of the i*PFY1*p-*yEGFP*-*CYC1* terminator cassette. The integrative plasmid hosting the device was named pINV1. Following pINV1 integration into the *URA3* locus, the device was characterised by flow cytometry after 6 hours of growth in SD glucose media without uracil and with 0 to 250 ngml^−1^ anhydrotetracycline (ATc) added (Fig. 5). The presence of TetR in the system represses the output of i*PFY1*p down to less than 5% of *PFY1*p output and the addition of ATc to saturating concentrations de-represses i*PFY1*p with the output reaching around 75% of *PFY1*p output. The inverter device has a dose-response to ATc which closely resembles a Hill function indicating the expected cooperative binding.

### Synthetic Orthogonal Repression of PFY1p using TAL Technology

TetR is a well-characterised, non-native transcriptional regulator suitable for yeast synthetic biology, but only offers one *wire* for gene network engineering. The inverter device characterised here, for example, could not function independently in cells already engineered with timer systems that utilise TetR [16]. For a scalable number of wires allowing orthogonal synthetic biology, new customisable transcriptional regulators are required that can themselves be re-engineered repeatedly to bind different DNA sequences. The recently-determined modularity of designer TAL effector (dTALE) proteins offers an attractive route to this as the DNA-binding motif of these proteins can be rationally and repeatedly re-programmed following simple motif-to-base recognition rules [29], [58].

In order to test whether TAL technology can be used to design and build custom transcriptional regulators that can orthogonally repress a promoter of choice, TAL proteins were designed to bind directly and specifically to the two different core promoter regions of *PFY1*p and i*PFY1*p. When choosing targets, similar sequences existing within the *S. cerevisiae* genome were identified and the binding scores of the dTALEs to these regions as well as to their targets were assessed. The dTALE targeting *PFY1*p had a target binding score of 3.37 and the highest affinity off-target site identified in the yeast genome, excluding the native *PFY1*p, was a sequence within *BAG7* with a score of 17.1. For i*PFY1*p these scores were 4.20 and 22.22 respectively with the highest affinity off-target site found being 213 bp 5′ of *ARR2*. As a further measure of orthogonality, a third TAL protein with a scrambled targeting sequence was also designed. To generate these dTALE transcriptional repressor proteins, we customised the modular TALE assembly kit [59] to yield modified TAL proteins whose expression is induced by the addition of galactose. The sequences generated using this system encode TAL proteins that lack the native activation domain and have both the native C-terminal, and an additional N-terminal, nuclear localisation signals. The TAL orthogonal repressors (TALORs) generated using the customised TALE kit encoded a *PFY1*p binding protein, P-TALOR, an i*PFY1*p binding protein, I-TALOR, and a scrambled DNA-binding protein, S-TALOR. The assembled TALOR-containing plasmids were each transformed into two yeast strains; one with pSV-*PFY1*p integrated at the *URA3* locus and one with pSV-i*PFY1*p at the *URA3* locus.

**Figure 6:**
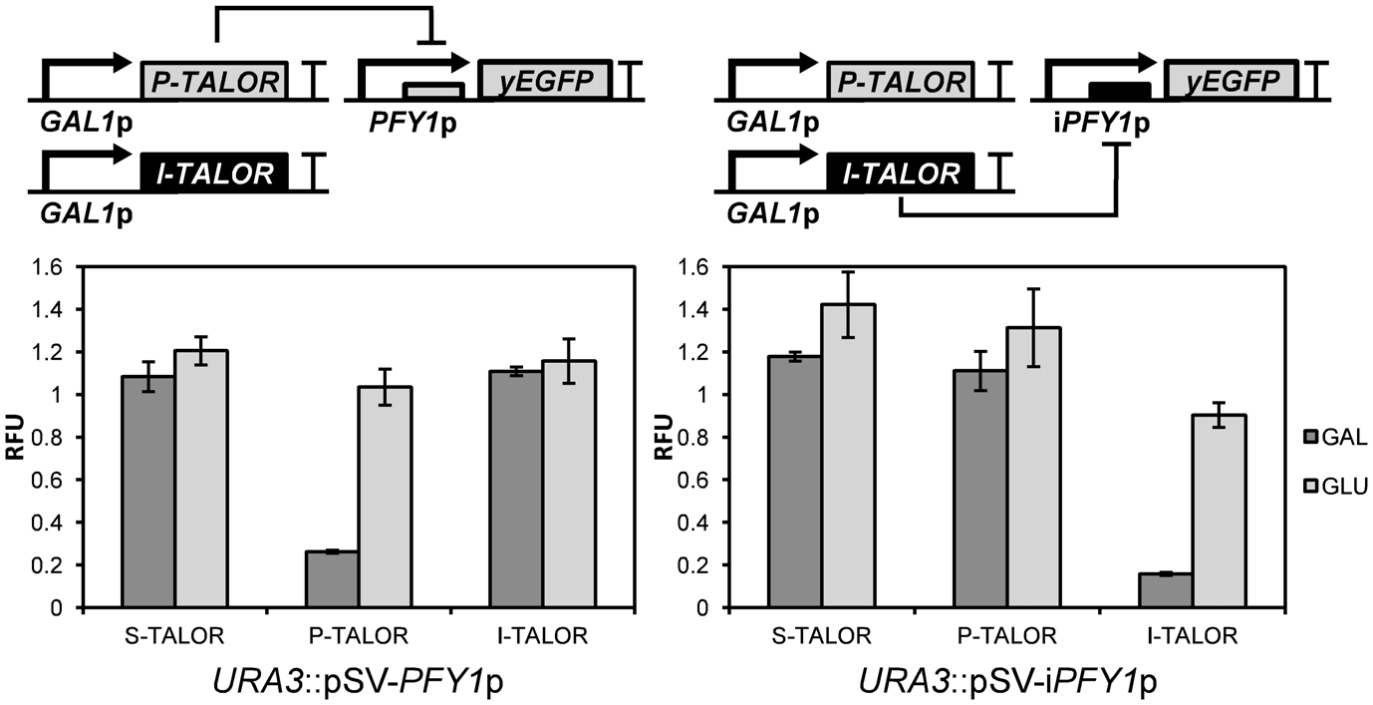
The effect of TALOR expression on promoter output. Fluorescence outputs of *PFY1*p and i*PFY1*p with and without induction of the expression of scrambled sequence-targeted S-TALOR, *PFY1*p-targeted P-TALOR and i*PFY1*p-targeted I-TALOR. Fluorescence is displayed in relative fluorescence units (RFU) and represents the mean average of geometric mean values as a proportion of a *PFY1*p control value for *PFY1*p output measurements (shown on the left graph) and as a proportion of an i*PFY1*p control value for i*PFY1*p output measurements (shown on the right graph). Error bars represent 1 standard deviation from the mean. GAL represents cells grown in galactose-containing media in which TALOR expression is induced and GLU represents cells grown in glucose-containing media in which TALOR expression is repressed. The gene circuit diagrams show the interaction between P-TALOR expression constructs and the *yEGFP* expression constructs.

Each TALOR-plasmid containing strain was characterised by flow cytometry after growth in 2% glucose or 2% galactose SD media without uracil and histidine for 16 hours (Fig. 6). With galactose induction, the two promoter-specific TALORs, P-TALOR and I-TALOR repressed their targeted promoter's output by 74% and 84% respectively, compared to identical strains with no TALOR plasmid. The control S-TALOR did not affect expression, as expected. Despite their target recognition sequences sharing a 9 bp homologous region (Fig. 4), these two TALORs only showed repression of the specific promoters that they had been targeted to. This work therefore demonstrates custom-targeted regulation by TALORs that are orthogonal to one another.

## Discussion

Characterisation of *PFY1*p, by both *in vivo* and *in silico* methods, has shown here that it has a stable constitutive output and that it is amenable to both random and designed sequence changes that alter the output strength and the regulation of the promoter. For these reasons, *PFY1*p could be described as an engineerable *backbone* biological part, suitable for rational customisation and rapid diversification to yield essential new biological parts. The promoter has a compact size, minimal natural regulation and a physiologically-relevant output that makes it useful for a variety of synthetic biology applications.

The generation of a *PFY1*p-derived synthetic promoter library yielded a set of constitutive promoters displaying a wide range of output levels (Fig. 3). A previous constitutive promoter library based on the *TEF1*p displayed a greater range of outputs, due to the native promoter being very strong. However this library, made through mutagenic PCR, delivers promoters longer than 400 base pairs in length that have on average greater than 95% sequence homology to the natural promoter [21]. Multiple use of members of this library within the same cell would pose a concern given yeast's proficiency in homologous recombination of similar DNA sequences. Members of our library, in contrast, are less than 200 bp in length and have less than 75% sequence homology (Fig. 4). Other synthetic promoter libraries described in yeast have been TATA-box based regulated promoters, containing specific elements such as sequences that promote transcription of glycolytic genes [20] or sequences that give expression only in the presence of galactose [16]. The ability to select a promoter with a predictable tuned output that is as close as possible to a desired value is extremely important in the design and construction of synthetic gene circuits [16] so all of these libraries provide valuable tools for synthetic biology. As with all such promoter libraries, validation of selected promoters in the intended genetic context is recommended prior to use to account for any context-related issues that may affect function.

A new class of user-targeted transcriptional repressors has been made available to synthetic biology by the demonstration that dTALEs can be designed and constructed to bind to and repress the output of promoters (Fig. 6). Although the TALORs generated in this work already repress transcription from their targeted promoter by around 80%, this repression could be further enhanced by designing two TALORs per promoter, possibly enabling co-operative binding. Fusion of functional protein domains that aid repression, such as DNA methylation domains, histone recruitment domains or the Krüppel-associated box repressor domain [60] may also be a promising way of increasing repression efficiency.

The use of TALORs targeted to promoters identical in sequence to those in the host cell raises potential problems in achieving regulation that does not affect native cellular processes that rely on the promoter in question. Although no phenotypic effects were observed when expressing P-TALOR in YPH500 cells, it is likely that PFY1 expression will have been affected as P-TALOR can bind directly to its core promoter. For this reason TALORs with a high affinity for off-target sequences within the host genome or an introduced synthetic network should be avoided. As the characterisation of binding efficiencies of TAL proteins to different sequences continues, aided by recent structural studies that elucidate their DNA-recognition mechanisms [61], [62], there may in future be additional criteria for what constitutes a desirable or undesirable target sequence. The coupling of TALORs with synthetic promoters, such as i*PFY1*p or promoters from a synthetic library, is therefore advantageous as it provides a variety of sequences from which to choose a target site and increases the orthogonality of the system by reducing the chance of TALORs targeting natural sequences and affecting endogenous gene expression.

The large amount of existing high-quality data relating to natural biology in *S. cerevisiae* is a valuable resource for identifying parts suitable for synthetic biology. While others have used such data to identify regulatory parts for use in synthetic networks, we used these data here to instead select promoters with constitutive behaviour into which we could rationally engineer synthetic regulation through repression. The well-characterised TetR regulator provides a sequence-specific, non-native part that represses transcription initiation through tight binding at the core promoter. With this we were able to create a new inverter device, giving the Boolean function NOT (Fig. 5). This part is valuable for further design of logic devices such as memory switches and counters, but the use of TetR as a wire in this design complicates interfacing it with existing or future devices and systems that also use TetR. As an answer to this problem, TALORs offer orthogonal repression that can be repeatedly reprogrammed, and in theory offer a limitless number of wires. Already designs exist for synthetic systems that would require multiple wires, such as adaptive learning systems that require multiple memory switches [63] and theoretical even-number repressilators [64]. The strategy presented here gives multiple synthetic promoters with diverse core sequences and, if the TALOR system proves to be scalable, the technology to regulate each independently. Therefore, with the combination of the synthetic promoter libraries and TALORs, any limits on the complexity of synthetic networks due to the lack of orthogonal regulated promoters would be effectively removed.

With projects such as the commercialisation of yeast-derived artemisinic acid and the synthesis of re-factored yeast chromosomal arms having a high impact in both the synthetic biology and wider communities it is clear that synthetic biology in yeast is growing in importance [12], [17], [65], [66]. The techniques used here to engineer regulation and tunable expression into *PFY1*p show that the strategy of diversifying and re-engineering existing parts to provide new functions is a valid and useful way to expand the toolbox for yeast synthetic biology.

## Materials and Methods

### Strains and Growth Conditions

The bacterial strain used for cloning was *E. coli* DH10B (Life Technologies). The yeast strain used for promoter characterisation was *S. cerevisiae* YPH500 [49]. DH10B was grown shaking at 200 rpm at 37°C in Luria Bertani broth. Ampicillin was added to media for selection where appropriate to a final concentration of 100 µgml^−1^. YPH500 was grown shaking at 200 rpm at 30°C in YPD broth, synthetic complete (SC) broth or synthetic drop-out (SD) broth lacking uracil, histidine or both [67]. Oligonucleotides were purchased from Integrated DNA Technologies and enzymes were purchased from New England BioLabs.

### Plasmid and Strain Construction

#### 1. Construction of pSV-*PFY1*p

A cassette of the *yEGFP* coding sequence, along with 7 bp of the 5′ UTR (Kozak sequence) and a 225 bp 3′ *CYC1* terminator sequence followed by a *Pvu*II site, was PCR amplified from the previously described pTVGI [16] using primers SV004 (5′- AACTACTAGTTATTAAAATGTCTAAAGGTGAAGAATTATTC-3′) and SV005 (5′- CCGCGCGTTGGCCGATTC -3′) to add a 5′ *Spe*I restriction site. The 189 bp region upstream of the *PFY1* CDS, from the −200 to the −12 position relative to the translational start site, has previously been shown to give maximum expression and was amplified from the *S. cerevisiae* YPH500 genome by colony PCR [47]. The amplification was performed using primers SV001 (5′- CAGCTGAATTCTGTGTGGGAGGTTTTACCATG-3′) and SV002 (5′- CTTCACCTTTAGACATTTTAATAACTAGTAGTTGGGTTTATGTTGTGTATG-3′), which added 5′ *Pvu*II and *Eco*RI sites, and a 3′ 29 bp homology to the 5′ end of the yEGFP PCR amplification product. The two PCR products were gel purified and fused by overlap extension PCR (OE-PCR) [68] with primers SV001 and SV005 to produce an 1183 bp product which was cloned into the yeast integration vector pRS406 as a *Pvu*II fragment to construct pSV-*PFY1*p [49].

#### 2. Construction of pSV-*ADH1*p, pSV-*BIO2*p, pSV-*CHO1*p, pSV-*CIT2*p and pSV-*CYC1*p

Promoter regions were amplified from YPH500 genomic DNA by colony PCR with primers that added a 5′ *Spe*I restriction site and a 3′ *Eco*RI restriction site to each amplified sequence. The amplified DNA was then cloned into pSV-*PFY1*p as a *Spe*I/*Eco*RI fragment, replacing *PFY1*p. The *ADH1*p sequence used was the 712 bp region 5′ of the *ADH1* CDS, from the −720 to the −9 position relative to the translational start site and was amplified using primers SV014 (5′- GCCGCCGAATTCGATATCCTTTTGTTGTTTCCG-3′) and SV015 (5′- GCCGCCACTAGTAGATAGTTGATTGTATGCTTGGT -3′). This region contains the −664 Rap1 UAS but not the 1006 Zap1 binding site, which is involved in promoter repression [69].

The *BIO2*p sequence used was the 383 bp region 5′ of the *BIO2* CDS, from the −391 to the −9 position relative to the translational start site as this region encompasses the known regulatory sites of the promoter [70]. The primers used to amplify *BIO2*p were SV006 (5′-GTTAGAGAATTCTAGTCATGTCGAGATGACTCG-3′) and SV007 (5′-GCCGCCACTAGTAAATTGAAAATAATCGGCTAAG-3′). The *CHO1*p sequence used was the 260 bp region 5′ of the *CHO1* CDS, from the −268 to the −9 position relative to the translational start site and was amplified using primers SV008 (5′-GCCGCCGAATTCCACTCCTTCTCAATGTGTG-3′) and SV009 (5′-GCCGCCACTAGTATATAGTTTTATTTTTGTTT-3′). This sequence has been shown to contain the two UAS regions required for promoter regulation [71]. The *CIT2*p sequence used corresponds to the largest promoter sequence used in a previous study of CIT2 regulation and consisted of the 992 bp region 5′ of the *CIT2* CDS, from the −1000 to the −9 position relative to the translational start site [72]. The primers used to amplify this region were SV010 (5′-GCCGCCGAATTCGACCAATGTTAATGA-3′) and SV011 (5′-GCCGCCACTAGTTTACTAGTATTATTAAAACA-3′). The *CYC1*p sequence used was the 420 bp 5′ of the CYC1 CDS, from the −432 to the −13 position relative to the translational start site. Previous studies indicate that this region contains all known regulatory elements of *CYC1*p [73], [74]. The primers used to amplify *CYC1*p were pSV016 (5′-ATTCAGGAATTCGGTAACAGTATTGATGTAAT-3′) and pSV017 (5′-GCCGCCACTAGTGTGTGTATTTGTGTTTGTG-3′).

#### 3. Construction of the *PFY1*p Synthetic Promoter Library

The *PFY1*p synthetic library was generated using a modified version of the synthetic promoter library technique [55], [56]. Partially-overlapping complementary oligonucleotides TES01 (5′-GCTCAGTTGACCCTTTCTCNNNNNNNNNNNNNNNNNNNNNNNNNNNCCTTGAGAAAAGAT-3′) and TES02 (5′-GACATTTTAATAACTAGTAGTTGGGNNNNNNNNNNNNNNNNNNNNNATCTTTTCTCAAGG-3′) were annealed and extended using Klenow polymerase to obtain 106 bp double-stranded DNA fragments. This library was inserted into the core promoter in the pSV-*PFY1*p plasmid using the CPEC method as previously described [57], with oligonucleotides TES11 (5′-GAGAAAGGGTCAACTGAGC-3′) and TES12 (5′-CCCAACTACTAGTTATTAAAATGTC-3′) being used to amplify the pSV vector. Resulting bacterial colonies from a single LB agar plate were pooled and plasmid DNA was extracted and integrated into the *URA3* locus of YPH500. Eight selected yeast colonies were screened for green fluorescence by flow cytometry to identify a single null-output promoter mutant. The null-output promoter was amplified by colony PCR using oligonucleotides SV001 and TES14 (5′ CGGTACCAAGCTTACTCGAG-3′). This was sequenced and incorporated into the pSV vector using restriction enzyme cloning to yield pSV-*PFY1*p.null. The synthetic promoter library technique method using CPEC and integrating into yeast as described above was then repeated at a larger scale and using the pSV-*PFY1*p.null plasmid as the template vector. This gave ∼10^5^ bacterial colonies over 16 LB Amp agar plates and then ∼10^3^ yeast colonies over 12 SD minus uracil agar plates. 275 colonies were picked and used to inoculate YPD media in 96-well plates, with growth and flow cytometry measurement of yEGFP expression of these cells performed as described below. 48 colonies that gave yEGFP expression over a graded-range were confirmed as single-integrations into the *URA3* locus and were sequenced following colony PCR using oligonucleotides SV001 and TES14. From this pool, 36 were selected for the final promoter library and were re-characterised in triplicate as described below.

#### 4. Construction of pSV-*iPFY1*p and pINV1

The promoter region of the *PFY1*p sequence of pSV-*PFY1*p was altered to introduce tandem Tn10 Tet operator sites (*tet*O_2_) within the core sequence using oligonucleotides TES05 (5′- CATTCCCTTCTTAAAACAACTGTCCCTATCAGTGATAGAGATCTCCCTATCAGTGA-3′ and TES06 (5′- TAGACATTTTAATAACTAGTAGTTGGGTTCTCTATCACTGATAGGGAGATCTCTATCA-3′). These partially-overlapping complementary oligonucleotides were annealed and extended using Klenow polymerase as above. The CPEC method was then utilised again to rapidly produce pSV-*iPFY*p, with amplification of the vector performed using TES07 (5′- CAGTTGTTTTAAGAAGGGAATG-3)′ and TES08 (5′-ACCCAACTACTAGTTATTAAAATGTCTA-3′). Plasmid pINV1 was constructed using the Gibson method [75] to assemble three sections into an ordered plasmid; *TEF1*p was amplified from pTVGI [16] using TES18 (5′-CTAATCTAGACATTTTAATAACCTAGGAAACTTAGATTAGATTGCTATGCTTTC-3′) and TES19 (5′-CGATTCATTAATGCAGCTGGAATTCCACACCATAGCTTCAAAATGTTTCTAC-3′), TetR plus the *ADH1* terminator was amplified from pTVGI using TES16 (5′-CATGGTAAAACCTCCCACACACCCGGGAATTGGAGCGACCTCATGCTATACC-3′) and TES17 (5′-GCAATCTAATCTAAGTTTCCTAGGTTATTAAAATGTCTAGATTAGATAAAAG-3′), and pSV-*iPFY1*p was linearised using TES15 (5′-CATGAGGTCGCTCCAATTCCCGGGTGTGTGGGAGGTTTTACCATGATTTTTGG-3′) and TES20 (5′-CATTTTGAAGCTATGGTGTGGAATTCCAGCTGCATTAATGAATCGGCCAACGCG-3′).

#### 5. Construction of TALORs

Custom TALORs were constructed using the TALE-NT kit (Addgene) [59] and a new destination plasmid (which we have named pTAL5) which was modified from the pTAL3 plasmid supplied. The pTAL5 plasmid was constructed using the Gibson method to assemble four sections into an ordered plasmid; *GAL1*p* [76] was amplified from pTVGI [16] using TES22 (5′-CTTACCGCGGAGACATATCGATGAATTCGAAGTACGGATTAGAAGCCGCCG-3′) and TES23 (5′-GGAGGAAGCCATTGTTGAACCTAGGCGGGTTTTTTCTCCTTGACGTTAAAGTATAG-3′), the TALEN destination protein coding sequence minus the FokI domain was amplified from pTAL3 [59] using TES24 (5′-CGTCAAGGAGAAAAAACCCGCCTAGGTTCAACAATGGCTTCCTCCCCTCC-3′) and TES25 (5′-CGAAGAATTGTTAATTAAGAGCTCTTATTAAGATATCGGATCCGGGAGGCCGCC-3′), the *ADH1* terminator was amplified from pTVGI using TES26 (5′-CCGGATCCGATATCTTAATAAGAGCTCTTAATTAACAATTCTTCGCCAGAGG-3′) and TES27 (5′-GGAAATTGTAAGCGTTAATCCCCGGGAATTGGAGCGACCTCATGCTATACCTGAG-3′), and the pTAL3 vector backbone was linearised using TES28 (5′-GCATGAGGTCGCTCCAATTCCCGGGGATTAACGCTTACAATTTCCTGATGC-3′) and TES21 (5′-CTTCTAATCCGTACTTCGAATTCATCGATATGTCTCCGCGGTAAGTTCGTACG-3′).

To design *PFY1*p- and i*PFY1*p-binding TALORs, the core promoter sequence of each (from the Reb1P-binding site to the 5′UTR) was entered into the single TALE targeter tool (http://boglab.plp.iastate.edu/node/add/single-tale/) [59] to derive predicted high affinity target sites and their corresponding RVD sequences (Fig. 6). The selected DNA site specific to *PFY1*p was 5′-ACACAACATAAACCCAACT-3′ and is bound by the TALE RVD arrangement NI-HD-NI-HD-NI-NI-HD-NI-NG-NI-NI-NI-HD-HD-HD-NI-NI-HD-NG. This sequence was assembled as previously described [45], but using pTAL5 as the destination plasmid in order to yield galactose-inducible P-TALOR. The selected DNA site for i*PFY1*p was 5′-AGAGAACCCAACTACT-3′ and is bound by NI-NN-NI-NN-NI-NI-HD-HD-HD-NI-NI-HD-NG-NI-HD-NG which was constructed as I-TALOR. The TALE targeter tool assigned each TALOR a binding affinity score and the Tal Effector Site Finder tool (https://boglab.plp.iastate.edu/node/add/talef) [59] was also used to assess the binding affinity to off-target genomic regions of similar sequences identified by BLASTn (http://blast.ncbi.nlm.nih.gov) [77]. A scrambled arrangement of RVDs was used to construct the control S-TALOR, which has an RVD order of NI-HD-NG-NN-HD-NI-NI-HD-NG-NI-NI-NN-NI-NN-NI-NI-HD-HD-NG and would target 5′-ACTRCAACTAARARAACCT-3′, a sequence which does not occur in the complete GenBank database. Plasmids were transformed into YPH500-derived strains via the lithium acetate/single-stranded carrier DNA/polyethylene glycol method [78].

### Chromosomal Integration of pSV plasmids into the *URA3* Locus of YPH500

For each plasmid to be integrated into the YPH500 genome, 200 ng of plasmid DNA was linearised into blunt ended fragments by restriction digest with *Stu*I, cleaving 437 bp into the 803 bp *URA3* CDS and purified using a PCR purification kit (Qiagen). The DNA was then transformed into the yeast cells via the lithium acetate/single-stranded carrier DNA/polyethylene glycol method [78]. Chromosomal integrants were isolated by selection on SD uracil-free media to enrich colonies in which *URA3* had been restored. Single integration events were confirmed by colony PCR screening amplifications using primers SV018 (5′-CAGATTGTACTGAGAGTGCA-3′) and SV019 (5′-TCCTTACGCATCTGTGCGGT-3′) to indentify multiple integrants (1172 bp band), and primers SV001 and TES14 (5′-CGGTACCAAGCTTACTCGAG-3′) to confirm integration of all constructs with PFY1-based promoters and the yEGFP sequence. All integrant strains used were confirmed as having a single integration event at the *URA3* locus.

### Characterisation of Promoters by Flow Cytometry

Yeast cultures with expression constructs integrated at the *URA3* locus were grown in triplicate overnight in appropriate media and measured for optical density at 600 nm (OD_600_) using a POLARstar Omega plate reader (BMG). A volume of 1 ml was taken from each culture, spun down in a 5424 centrifuge (Eppendorf) and the pellet was re-suspended in SC media without a carbon source to a volume that normalised the OD_600_ to 2. In Costar flat-bottomed 96 well assay plates (Corning), each culture was used to inoculate appropriate media 1 in 10 up to a final volume of 200 µl. Plates were sealed with a Breathe-Easy sealing membrane (Sigma-Aldridge) and incubated in a Microtron incubator (Infors HT) at 30°C shaking at 710 rpm.

At appropriate time points, 20 µl samples were taken from each well and transferred to a separate microplate where distilled water was added up to a volume of 200 µl. Cells were measured using a FACscan flow cytometer (Becton Dickinson) with a 96-Well Automated Micro-Sampler (Cytek). Data was acquired using CellQuest software (Becton Dickinson) with wells being sampled on high flow rate for 20 seconds. Data was analysed using Cyflogic software (CyFlo) with a tight forward scatter/side scatter gate being applied to ensure a homogenous population size. Fluorescence values given represent the mean value of the geometric mean expression values of each replicate for that time point and standard deviation is also calculated from the replicate geometric mean values. Where coefficient of variation values are given for a promoter they represent the standard deviation of all replicate values divided by the mean of all replicate values. Where units of fluorescence are given as relative fluorescence units (RFU), values have had the fluorescence value of YPH500 control calls subtracted and are given as a proportion of *PFY1*p control cell fluorescence. All controls were grown in triplicate under the same conditions (except with the addition of uracil and/or histidine to SD media where appropriate to allow growth) and had fluorescence values measured on the same plates as the assayed cells.
